# Soft Palate Modification Using a Collagen Crosslinking Reagent for Equine Dorsal Displacement of the Soft Palate and Other Upper Airway Breathing Disorders

**DOI:** 10.1155/2019/9310890

**Published:** 2019-04-01

**Authors:** Stephanie Hunt, Jonathan Kuo, Fabio A. Aristizabal, Matt Brown, Abhijit Patwardhan, Thomas Hedman

**Affiliations:** ^1^Biomedical Engineering Department, University of Kentucky, Lexington, KY, USA; ^2^Crosscoat Medical, LLC, Lexington, KY, USA; ^3^School of Veterinary Medicine, University of California-Davis, USA

## Abstract

The mechanical properties of the soft palate can be associated with breathing abnormalities. Dorsal displacement of the soft palate (DDSP) is a naturally occurring equine soft palate disorder caused by displacement of the caudal edge of the soft palate. Snoring and a more serious, sometimes life-threatening, condition called obstructive sleep apnea (OSA) are forms of sleep-related breathing disorders in humans which may involve the soft palate. The goal of this study was to investigate the effect of injecting the protein crosslinker genipin into the soft palate to modify its mechanical properties for the treatment of equine DDSP with potential implications for the treatment of snoring and OSA in humans.* Ex vivo* experiments consisted of mechanical testing and a wind tunnel study to examine the effect of genipin on the mechanical properties, displacement, and vibration of equine soft palates. A pilot* in vivo* study was completed using DDSP and control horses to test the safety and effectiveness of injecting a genipin reagent into the soft palate. The wind tunnel testing demonstrated a greater than 50% decrease in transient deformation and a greater than 33% decrease in steady-state vibrations for all doses of genipin tested. Ultimate tensile stress, yield stress, and Young's modulus were higher in the genipin-treated distal soft palate specimens by 52%, 53%, and 63%, respectively. The pilot* in vivo *study showed a reduction of snoring loudness in all DDSP horses and elimination of DDSP in at least one of three horses. The difficulty of using a 1-meter-long endoscopic injection needle contributed to a consistent overinjection of the equine soft palates, causing excessive stretching (pillowing) and related degradation of the tissue. These* ex vivo* and* in vivo* results demonstrated reduced vibration amplitude and flaccidity and increased strength of genipin-treated soft palates, suggesting that genipin crosslinking could become an effective and safe treatment for soft palate related breathing abnormalities.

## 1. Introduction

Whether the result of repetitive insult or a congenital condition, a flaccid soft palate can be involved in upper airway obstruction and sound generation in animals and humans. Dorsal displacement of the soft palate (DDSP) is a naturally occurring soft palate disorder caused by displacement of the caudal edge of the soft palate, interfering with normal expiratory airflow through the nasopharynx (i.e., expiratory resistive breathing, as opposed to resistive breathing in human snoring and obstructive sleep apnea which involve resistance during inhalation), thus producing mechanical property deficiencies, snoring, and temporary obstruction of the airway [1]. The displacement can usually be self-corrected by the animal through swallowing or readjusting the head position; however, it can occur several times during a single training session or race causing interruptions to normal breathing and, consequently, a significant decrease in performance. Overall, DDSP is estimated to occur in 10-40% of competitive horses, especially Thoroughbreds and Standardbred racehorses [2–8]. Clinical symptoms consist of a loud gurgling or snoring noise, fluttering of the cheeks as air is diverted beneath the palate, exercise intolerance, or commonly described by trainers as “choking down” or “swallowing their tongue” [2]. Conservative treatments include tongue-tying during exercise, various types of bits, or the Cornell Collar. When conservative treatments fail, more invasive surgical procedures are completed, including the laryngeal tie-forward procedure, laser staphylectomy, myectomy, palatal sclerotherapy, or a combination of different procedures, which have a reported 58-78% clinical success rate [2–9] but without a consensus treatment of choice [10]. With some similarities, snoring is a condition that affects people of all ages, including 48% of men and 34% of women [11–13]. Another sleep-related breathing disorder, obstructive sleep apnea (OSA), is a more serious, and sometimes life-threatening, condition that occurs in approximately 4.2% of Americans aged 16 and older [14–18]. Both snoring and OSA are the result of obstructed airflow during inhalation due to abnormalities in the geometry of the air passages and the propensity for aberrant deformation of the soft palate [19, 20]. Current treatments for snoring and OSA include lifestyle changes, oral appliances, surgery, or minimally invasive procedures targeted at stiffening the soft palate [21, 22]. Many of the newer procedures rely to some extent on the formation of fibrotic scar tissue to stiffen the soft palate [20, 23]. This fibrotic tissue can be expected to exhibit inferior mechanical properties and a decrease of structural integrity compared to normal tissues, leading to the eventual loss of some initial treatment-related benefits [20–29].

Genipin (GP) is a minimally toxic, protein crosslinker extracted from the* Gardenia jasminoides* plant. GP has been previously investigated with regard to its ability to modify the mechanical properties of annulus fibrosis tissue in the intervertebral disc [30, 31]. The study hypothesis was that an injection of a GP reagent into the soft palate would modify the mechanical properties of soft palate tissue and thus reduce aberrant deformation and vibration when subjected to physiological air flow, while increasing tissue strength. Further, it was hypothesized that these modifications to the soft palate tissue could decrease the severity of snoring and the likelihood of airway collapse, while also increasing the tissue's resistance to mechanical degradation. Rather than generating scar tissue, crosslink augmentation serves to stabilize the tissue, preserving microstructural and compositional integrity, increasing strength, stiffness, fatigue resistance, and the energy required to permanently deform (resilience) or fail (toughness) the tissue, in keeping with its demonstrated effect in other collagenous tissues [30–32]. The hypothesis was tested using equine soft palates in* ex vivo* studies including wind tunnel testing and mechanical testing and using normal horses and horses with confirmed DDSP in a small pilot* in vivo* study.

## 2. Methods

### 2.1. Wind Tunnel Study

**Figure 1:**
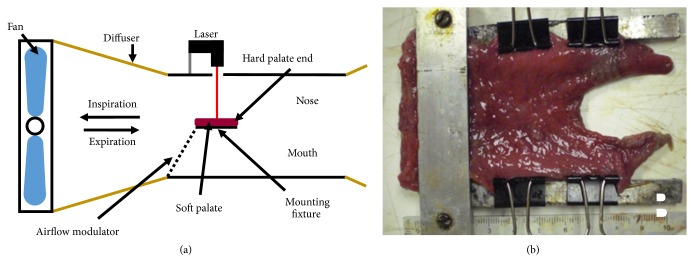
Wind tunnel apparatus. A wind tunnel was constructed to simulate simplified human or equine inhalation or exhalation airflow (a) consisting of a fan that blew air over the soft palate, an airflow modulator, and a laser micrometer to measure soft palate displacements. The soft palate was constrained (b) on the hard palate and lateral edges.

A simplified wind tunnel system was developed to quantitatively measure the displacement and vibration of soft palate specimens under simulated human inhalation (Figure 1(a)). Twenty-five equine soft palates were acquired from a local vendor and divided equally into five groups: untreated control, GP soaked, and 50 mM, 100 mM, and 150 mM GP injections. The GP soaked specimens and untreated controls served as baselines for maximum crosslinking treatment and for untreated tissue, respectively. A solution of 0.33 wt. % GP (330mg GP/100ml phosphate-buffered saline, PBS) solution was used to bathe soft palates to allow for maximal crosslinking in the soaked group. For the injection treatment groups, genipin was solubilized in a solution of PBS that included 40% dimethyl sulfoxide (DMSO) to increase the solubility of the crosslinker. For each concentration, two 1 ml bilateral injections were delivered 2 cm from the V-notch of the free (distal) edge of the soft palate (Figure 1(b)). All specimens were covered with PBS-soaked paper towels and placed in plastic bags on a rocker plate for 24 hours at room temperature to allow ample time for the GP to diffuse through the tissue and the crosslinking to take place. When GP reacts with primary amine groups, it generates a blue pigment that is visible to the naked eye and serves as a useful indicator of diffusion distance and density of crosslinking. After being allowed to react for 24 hours, the soft palate specimens were imaged so that the area coverage could be analyzed and the distribution of genipin estimated using ImageJ software (NIH). Preliminary testing investigated the effects of PBS-DMSO buffer-only treatment and 37°C incubation temperatures. Either condition in the absence of crosslinker resulted in excessive tissue degradation making mechanical testing problematic.

Soft palate specimens were mounted into a custom made C-shaped aluminum clamp that represented the support provided by the hard palate and lateral connective tissues, stabilizing the regions around the hard palate but allowing for free movement of distal tissue (Figure 1(b)). Each soft palate specimen was subjected to wind tunnel testing prior to treatment to serve as its own control. The wind tunnel system produced transient and steady-state wind flow up to 14 m/s (measured using a Celestron anemometer) over the mounted soft palate surface to simulate breathing conditions estimated from human tidal breath volume of 500 ml, period of 2 seconds, nasal cross-sectional area estimate from Smith [33]. Equine airway velocities may be considerably higher based on the findings of a study using an experimental data validated computational model [34], which showed peak expiratory velocity in the region of the epiglottis to be approximately three times higher. A laser micrometer (Keyence LK-081, Itaska, IL) was mounted above the sample to measure the displacement/vibration amplitudes and frequencies. National Instruments (NI) MAX software was used to capture 10-second intervals of data at 1 KHz in three regions of the soft palate: free edge (0.5 cm from edge), middle (1.5cm towards hard palate), and hard palate end (1.5cm closer to hard palate). The change in displacement amplitudes and vibration frequencies due to treatment were compared between groups. Additionally, three human cadaveric soft palates were similarly tested in the wind tunnel with two bilateral injections of 0.5 ml of the 100 mM genipin solution.

### 2.2. Mechanical Properties of Soft Palates

A study was completed to determine changes in the mechanical properties of the soft palate when treated with a buffered 50 mM GP solution. Each of twelve equine soft palates were divided into four sections cut parallel to the free edge producing two sections per lateral side, with each section subsequently cut into an hourglass shape and its dimensions measured. The sections were separated into three groups: control (untreated), buffer-only injection (0.5 ml of 0.9% saline with 50 mM sodium phosphate), and buffered genipin injection (0.5 ml of 50 mM GP in saline-phosphate buffer). Injections were done using a 25 mm, 22-gauge needle. The needle was inserted at an angle of approximately 30 degrees from the horizontal in the approximate center of the specimen, and the reagent was delivered with uniform pressure over five seconds as the needle was being extracted from the tissue. All sections were covered with saline-soaked paper towels and placed on a rocker plate for 12-15 hours at room temperature to allow time for GP diffusion and maximal crosslinking prior to testing.

Cyclical hysteresis, stress relaxation, and tensile loading to failure were evaluated using a materials test system (Test Resources 100R1000). The cyclical loading consisted of a 10 N preload combined with 20 cycles of ±3 mm deformation at a rate of 5 mm/s, allowing the cyclical hysteresis to be quantified. The hysteresis ratio was defined as the ratio of the 1st and 20th cycles, and the percent changes in hysteresis and hysteresis ratio from pretreatment to posttreatment were calculated. The stress relaxation test involved a 10 N preload followed by a five-minute hold in displacement to calculate stress relaxation and relaxation modulus. The changes in relaxation metrics before and after treatment were calculated. Finally, tensile load to failure began at zero tensile load and proceeded at a rate of 1 mm/s to failure, and ultimate tensile stress (UTS) and corresponding strain, Young's modulus (E), toe region modulus, yield stress and strain, toughness, and resilience were calculated. Outliers, determined by the three-sigma rule, were removed from the data sets. The averages and standard deviations for each parameter were calculated for the individual test groups. Any parameters with a percent difference greater than 30% between the buffer-only group and the GP group were noted. Statistical analysis (described below) was completed to compare the buffer-only group to the control group and to compare the buffer-only group to the GP group.

### 2.3. Pilot* In Vivo* Study

The pilot, IACUC approved,* in vivo* study served to determine preliminary safety and effectiveness of injecting equine soft palates with a buffered GP reagent. Sterile buffer solution—(45 mM 3-[4-(2-Hydroxyethyl)-1-piperazyl] propane sulfonic acid phosphate (EPPS-P) buffer, 8.5 pH, with 10% DMSO to enhance GP solubility)—was produced by autoclaving and sterile filtering (Corning, 0.20*μ*m), the solution prior to dispensing into sterile vials which were sealed until use within a laminar flow hood. The GP required for a 100 mM concentration was weighed out into sterile vials and sealed before being terminally sterilized by gamma irradiation (Steris, 25-50 kGy). Immediately prior to each horse's soft palate treatment, 2 mL of the buffered solution was added to one GP vial to produce 2 mL of the 100 mM buffered GP reagent. Based on the wind tunnel test results, this crosslinker concentration was essentially double the effective concentration such that an absence of clinical benefit in treating DDSP horses would suggest a failure of this mode of treatment, and the absence of serious morbidity or complications would signal that a safe concentration can be determined.

The safety phase of this study had three control horses, and the efficacy phase had three DDSP horses. Prior to the soft palate treatment, the efficacy phase horses underwent dynamic endoscopic examinations and breathing audio recordings to confirm DDSP diagnoses and to document snoring loudness and frequency. The dynamic endoscopic examinations were conducted by two different equine veterinarians, involving walk, trot, and canter exercise either on a treadmill or using a lunge line or while harness racing as permitted by the horse owners. Both of these tests were repeated after treatment at the same exercise levels to compare and quantify differences. All six horses were injected using a 1-meter-long transnasal endoscope by a single veterinarian with two 1 ml injections of 100 mM buffered GP solution in the 5:00 and 7:00 positions with the epiglottis occupying 6:00. Prior to the soft palate injections, the veterinarian thoroughly examined each horse and provided standing sedation determining the appropriate dose of intravenous detomidine and butorphanol based on the animal's weight. Also, approximately 10 to 20 mL of 2% lidocaine was sprayed onto the surface of the caudal margin of the soft palate through the injection port of the endoscope to induce topical local anesthesia. After treatment, the horses were monitored hourly for 72 hours for signs of distress or difficulty breathing. One week after treatment, the safety phase horses were euthanized (sedated with intravenous detomidine (0.02 mg/kg) and butorphanol (0.03 mg/kg) followed by a Fatal Plus (1 mL/4.5kg) injection) and their soft palates were harvested and immediately placed on dry ice for storage until further histological analysis. Each palate produced four samples from the treated regions of tissue as determined by the visual observation of the blue GP crosslinking coloration and two samples from an apparently untreated region. All biopsies were sectioned in the sagittal plane such that both the mucosal surfaces along with submucosal and muscular components were represented. All tissues were processed for paraffin infiltration, blocked, sectioned, stained with H&E, and examined microscopically. Observed microscopic changes were described as to the extent and grade of inflammation and fibroplasia using terms including minimal, mild, moderate, and marked.

The efficacy phase DDSP horses were returned to their owners 10-14 days after treatment. Ten-second intervals were selected from the 20-minute audio recordings and analyzed in the time domain, where gaps in the breathing patterns indicating potential airway blockages were noted. The audio recordings were also transformed into the frequency domain using the fast Fourier Transform (FFT). In the frequency domain, spectrogram graphs were created in Matlab (MathWorks) and analyzed using the green color channel in ImageJ to determine changes in amplitude intensities at various frequencies. High frequency range (500 to 4000 Hz-the Nyquist frequency) and low frequency range (0 to 100 Hz) changes were expected to correspond to palatal displacement generated whistling and snoring, respectively [26, 35]. The frequency domain data were filtered using a moving average filter. The areas under the frequency domain curve were calculated using the trapz function in Matlab to represent the spectral density of the audio file, and then the percent difference was determined to quantify a difference between the pretreatment and posttreatment amplitude files. Finally, a study by Franklin et al. discovered a peak in the 53±24 Hz range corresponding to an apneic soft palate displacement in DDSP horses [36]. Therefore, the frequency domain graphs were analyzed from 20 to 80 Hz before and after treatment to detect an apnea/displacement related peak.

### 2.4. Statistical Analysis

Statistical analysis was completed by first using the F-test to determine whether the sample variances were equal; if considered equal a Student's* t*-test was used for analysis and if the variances were not considered equal, a Mann-Whitney* U* nonparametric test was used. Statistical significance was considered between groups with *α*=0.05.

## 3. Results and Discussion

### 3.1. Wind Tunnel Study

**Figure 2:**
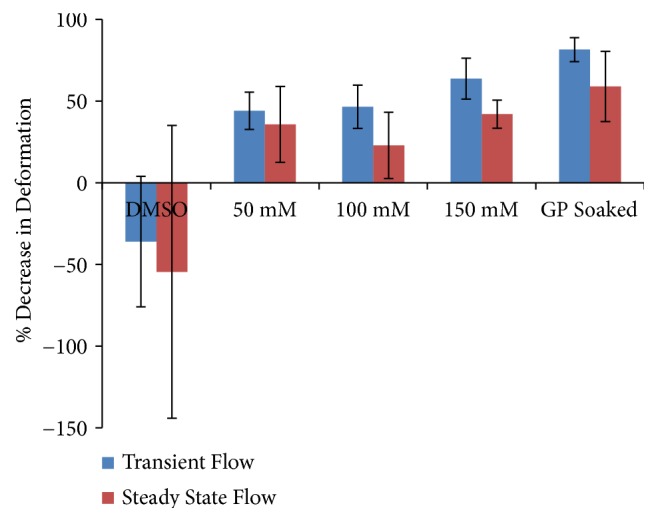
Soft palate deformations. Soft palate free end deformation change from pretreatment to posttreatment in both transient and steady-state flow. Data are mean±SD (n=5).

Percentage of tissue crosslinked varied by concentration of GP injected, the control (soaked) had 100% coverage and 50, 100, and 150 mM had approximately 40, 49, and 51% coverage, respectively. The largest amount of soft palate deformation was seen in the free edge so this area was the focus for analysis of the effect of crosslinking treatment. The percent reduction in transient and steady-state displacements is summarized in Figure 2. Treatment with all GP injection concentrations combined resulted in average decreases of 51.4% and 33.5% in deformation of the soft palate when subjected to transient and steady-state wind flow, respectively. As noted above, the limitations of this simplified wind tunnel model, especially with regard to the airflow associated with equine DDSP (possibly 3-fold higher velocities in expiration), may suggest that the effects of genipin protein crosslinking in this wind tunnel model may not accurately reflect the magnitudes of clinical effects in horses. Fourier analysis of wind tunnel data showed an average decrease in amplitude for all GP injected treatments of approximately 24% and an average increase in frequency of 14 Hz, though the results were not statistically significant. Samples that were soaked in GP had significantly dampened vibration during transient and steady-state wind flow compared to the control groups. This group demonstrated the effects when maximum crosslinking was achieved. Injections of genipin resulted in more localized crosslinking indicated by blue coloration extending approximately 2 cm from the site of injection with a trend of increasing areas of treatment resulting from increases in crosslinker concentration.

The human soft palate specimens free-end maximum transient deformations were reduced by an average of 32.4% and maximum steady-state deformations were reduced by an average of 19.6% compared to pretreatment testing. These pilot human wind-tunnel experiments demonstrated damping magnitudes similar to those in the equine studies.

### 3.2. Mechanical Properties of Soft Palates

UTS increased 52% (p=0.021) and ultimate tensile strain decreased 31% (p=0.006) in the genipin-treated group compared to the buffer-treated group. Yield stress increased 53% (p=0.017), and Young's Modulus increased 63% (p=0.026). The samples from regions more proximal to the hard palate demonstrated higher variability in treatment effects. Therefore, results are listed for all regions and distal regions only (near free edge of soft palate) in Table 1.

While the genipin treatment altered some of the elastic-plastic properties of the soft palate, it did not produce a significant effect on the resilience (the energy required to plastically deform the tissue) or the toughness (the energy required to permanently deform the tissue). Additionally, none of the results from the hysteresis test or the stress relaxation tests produced statistically significant differences between the genipin-treated and buffer-only-treated specimens. Thus, there was no demonstrated effect of the genipin crosslinker on the viscoelastic properties of the soft palate in this small study.

### 3.3. Pilot* In Vivo* Study

All six horses were successfully treated with two 1 mL transendoscopic injections of the genipin reagent in the 5:00 and 7:00 locations on the soft palate. A pillowing expansion of the hemi-palate was observed upon injection in each case. One safety phase horse was unintentionally given two 1.5 mL doses due to an error in calculating the priming volume in the needle and tubing. One efficacy phase horse experienced mild hypersalivation and a reduced appetite, which subsided within 24 hours.

**Figure 3:**
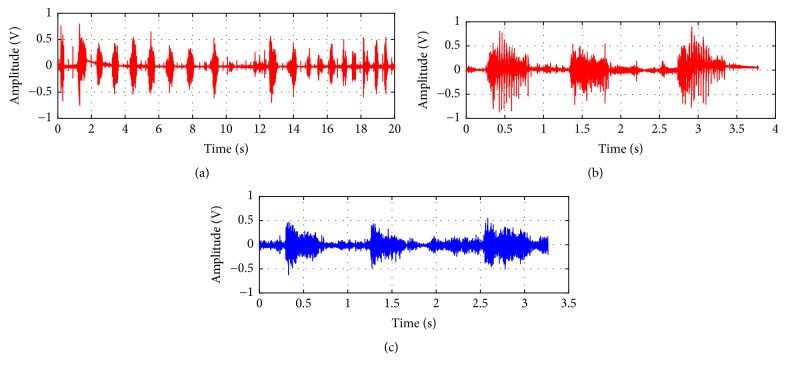
Typical time domain data. Representative time domain audio data showing (a) a typical pretreatment breathing gap and a comparison between pretreatment (b) and posttreatment (c).

**Figure 4:**
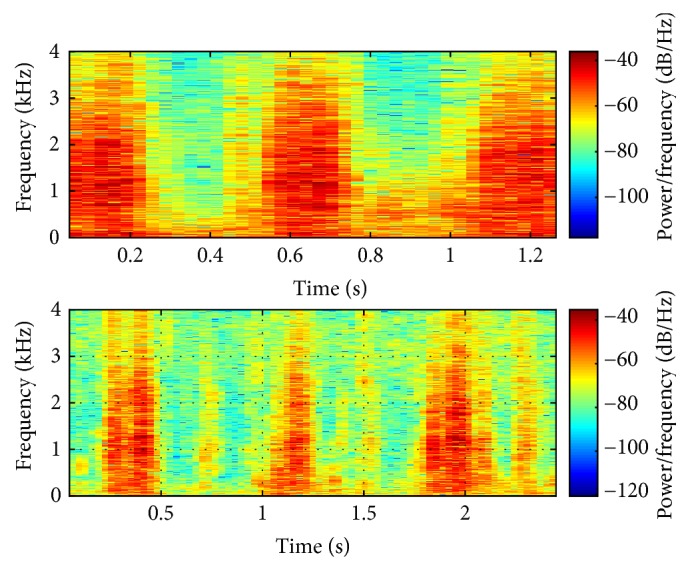
Typical audio spectrogram data. Representative spectrogram graphs demonstrating a reduction in high amplitude signal across all frequencies, especially in the high frequency range after treatment.

After returning home, two of the efficacy phase horse owners reported an improvement in the horse's breathing and a decrease in exercise intolerance. The other DDSP horse had intermittent improvement in the DDSP symptoms. Postinjection dynamic endoscopic evaluation revealed that the horse with intermittent improvement still had DDSP, while one of the horses showing marked improvements no longer had DDSP. The other horse showing an absence of DDSP symptoms was not evaluated by dynamic endoscopy due to logistical issues. All three efficacy phase horses had a reduction in time domain audio amplitude peaks, as well as a more uniform breathing pattern, including elimination of gaps in breathing (typically reflecting a swallowing event [37]), in the posttreatment graphs compared to pretreatment (Figure 3). Similarly, spectrograms showed reductions in amplitudes across a variety of frequencies with treatment, with the red color representing high amplitudes and the blue color representing low amplitudes (Figure 4). In particular, there was a decrease in the high amplitude signals in the high frequency range for all three horses and in the low frequency range for two of the horses. Overall, there was an average 39% reduction in the area of high amplitude red coloration.

**Figure 5:**
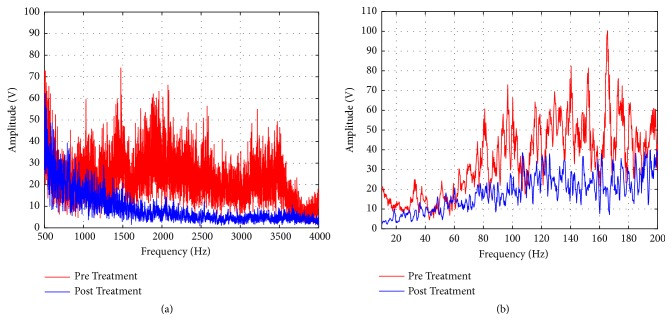
Typical frequency domain data. (a) Representative high frequency domain graph from 500 to 4000 Hz showed a decrease in posttreatment amplitudes related to a reduction in soft palate whistling sounds. (b) Representative low frequency domain graph from 0 to 200 Hz showed a decrease in posttreatment amplitudes related to a reduction in loudness of snoring sounds and vibrations.

Spectral density in the high frequency range (>500Hz) was reduced 4-fold in one DDSP horse after treatment (Figure 5(a)) and reduced slightly less than 100% in another horse, while spectral density in the low frequency range (<200Hz) was reduced 27-62% in the three horses (Figure 5(b)). The horse that did not demonstrate a reduction in high frequency amplitudes was the horse found to still exhibit DDSP during the dynamic endoscopic examination. Reduction in high frequency whistling noises could therefore be associated with the elimination of palatal displacement in the equine model. These apnea-related audio results were also confirmed by the analysis of the Franklin et al. DDSP frequency range [36]. Each of the horses demonstrated peaks in this range before treatment, but after treatment, the peaks remained only in the horse found to still exhibit DDSP. Similarly, the consistent reduction in low frequency audio amplitudes in all three horses after treatment corresponded to the owner reported reduction in snoring/breathing loudness.

**Figure 6:**
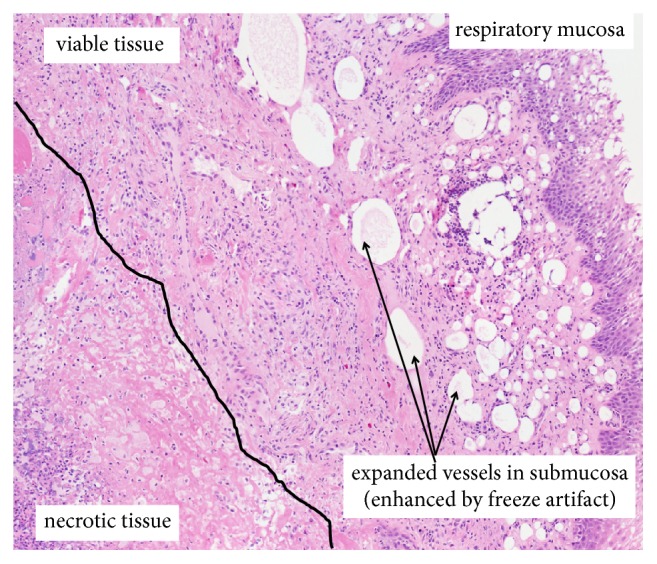
Histological observations from safety phase soft palates. There were moderate-to-extensive regions of tissue disruption and patchy necrosis with mild-to-moderate inflammatory and fibroplastic responses in viable tissue in both treated and untreated regions of the soft palate, believed to be due to the consistently observed excessive expansion of the soft palate (pillowing) during the injections.

Histological analysis of the safety phase soft palates showed no specific trends in the severity or extent of inflammatory infiltrates with regard to the genipin treatment at 7 days after treatment. However, in all treated and untreated samples, there were moderate-to-extensive regions of tissue disruption (tearing) toward the mucosal surface and patchy necrosis toward the middle of the soft palate (Figure 6). Additionally, biopsies in these more central regions showed mild-to-moderate inflammatory and fibroplastic responses in viable tissue. By the presence of tissue disruption and inflammatory responses in both the treated and untreated samples, it can be presumed that the delivery method was responsible for the fibrotic tissue formation. The rapid (1-2 seconds) injections were associated with a consistently observed excessive expansion (pillowing) of the soft palate tissues. The outer regions of the tissue would have undergone the most strain corresponding to the regions of tissue tearing. These histologic observations suggest that future injections should be done with a decreased reagent volume and a slower rate of injection to promote flow and diffusion of the reagent through the tissue rather than creating a sudden bolus of reagent in the middle of the tissue. It is also a consideration that the overstretching of the tissue and subsequent healing response may have contributed to the overall success of the treatment in the DDSP horses. However, laser palatoplasty induced inflammation and fibrosis that was observed in a study by Alkabes et al. [38] resulted in a significant decrease in soft palate elastic modulus. This finding may suggest that the fibrosis and inflammation resulting from the tissue stretching (at the time of injection) in the current study may have reduced the palatal stiffening effects seen in the efficacy phase horses.

The histological analyses were conducted on the safety phase horses exclusively at 7 days after treatment. This constitutes a limitation of the present study considering that other similar soft palate treatment studies evaluated changes to the treated tissues from 2 to 45 days [38] and from 2 weeks to 6 months [39] after treatment. In particular, it would be valuable to have audio and dynamic endoscopic data from efficacy phase horses at later time-points corresponding to histological data from sacrificed horses. Alkabes et al. [38] found that edema and inflammation decreased from day 2 to day 45. Therefore having later time-points in subsequent studies may make it possible to deduce the relative contribution of tissue stretching induced inflammation and fibrosis to the increased palatal stiffness and reduced palate displacements observed in this first study. Likewise, minimization or elimination of tissue overstretching during injection in future studies would enable assessment of the genipin crosslinking effect alone in the prevention of palate flipping, breathing disruption, and snoring loudness. A longer period of postinjection testing would also be important to assess the durability of these presumed permanent covalent bond induced effects.

The pilot* in vivo *study demonstrated that injecting genipin is feasible and effective in reducing snoring loudness and preventing soft palate displacements. The treatment was determined to be safe; however, excessive expansion of the soft palate tissue during the injection should be avoided. Aberrant injections would be less likely if this treatment was applied to human soft palates because of the difference in accessibility. All of the DDSP horses exhibited a reduction in snoring loudness, and at least one of the DDSP horses was found to no longer exhibit DDSP. An injectable treatment utilizing protein crosslinking may also provide the added potential benefits of being easier to administer, less expensive, more durable, and less harsh compared to current treatments and emerging minimally invasive approaches for treating equine DDSP.

## 4. Conclusions

The genipin reagent injection demonstrated a reduction in soft palate deformation and vibration amplitude and an increase in tissue strength, stiffness, and stability based on the results from the mechanical testing and wind tunnel studies. Pilot* in vivo* studies demonstrated that with appropriate delivery to the soft palate tissues the treatment could be safe and effective in reducing snoring loudness and preventing soft palate apneic displacements. These results suggest that genipin-based treatments should be further evaluated for equine-scaled air flow and duration of effect as it could become an effective and safe treatment for equine DDSP and other upper airway breathing disorders that involve a flaccid soft palate.

## Figures and Tables

**Table 1:** Mean differences between genipin-treated specimens (50 mM) compared to buffer-only treatment for each of elastic-plastic and viscoelastic parameters.

Parameter	Distal samples only	All samples
Cross-sectional area	11.95% decrease	20.49% decrease
Ultimate tensile stress	51.67% increase*∗*	31.20% increase
Ultimate tensile strain	32.89% decrease	30.88% decrease*∗*
Yield strain	34.57% decrease	30.65% decrease
Yield stress	53.04% increase*∗*	42.69% increase*∗*
Young's modulus	63% increase*∗*	56.02% increase*∗*
Resilience	6.43% decrease	27.51% decrease
Toe region modulus	118.34% increase	118.33% increase
Toughness	11.99% increase	9.71% increase
1st cycle hysteresis	10.31% increase	23.41% increase
Change in hysteresis	1.08% decrease	14.88% increase
Hysteresis ratio	30.20% increase	23.24% increase
Stress relaxation	4.09% increase	9.19% increase

*∗* Statistically significant differences (p < 0.05).

## Data Availability

The wind tunnel, mechanical testing, and pilot* in vivo* testing data used to support the findings of this study are available from the corresponding author upon request.
